# Stent with radioactive seeds placement for obstructive hilar cholangiocarcinoma: comparison between unilateral and bilateral placement

**DOI:** 10.20452/wiitm.2025.17984

**Published:** 2025-10-01

**Authors:** Gang Wang, Xue Wang, Yi ‑Bing Shi, Ying Zhu

**Affiliations:** Department of Radiology Xuzhou Central Hospital Xuzhou China; Medical Image Center, Xuzhou Mine Hospital, Xuzhou, China

**Keywords:** bilateral, hilar cholangiocarcinoma, radioactive seeds, stent, unilateral

## Abstract

**INTRODUCTION:**

Biliary stenting with radioactive seed placement has become a frequently applied palliative intervention for patients with obstructive hilar cholangiocarcinoma (HC). Despite its increasing use, evidence comparing unilateral and bilateral stenting with radioactive seed placement remains limited.

**AIM:**

This study aimed to compare clinical effectiveness and safety of unilateral and bilateral stenting with radioactive seed placement in patients with obstructive HC.

**MATERIALS AND METHODS:**

A retrospective review of consecutive patients treated with a stent with radioactive seed placement for obstructive HC between January 2022 and December 2024 was conducted. The patients were categorized into the unilateral or bilateral groups based on the stent placement approach. Technical success, clinical response, stent patency, overall survival, and complications were compared between the groups.

**RESULT:**

A total of 89 patients were analyzed, including 47 in the unilateral group and 42 in the bilateral group. Primary technical success was achieved in 91.5% and 95.2% of the patients, respectively (*P* = 0.68), while the secondary success rate was 100% for both groups. Clinical success rates were comparable (89.4% vs 90.5%; *P* >0.99). Median (interquartile range [IQR]) stent patency was 220 (160–256) days for unilateral placement and 210 (166–216) days for bilateral placement (*P* = 0.79). Median (IQR) overall survival was 255 (178–369) and 242 (175–362) days, respectively (*P* = 0.79). Incidence of cholangitis (10.6% vs 9.5%; *P* >0.99) and bleeding (4.3% vs 4.8%; *P* >0.99) did not differ between the groups.

**CONCLUSION:**

Both unilateral and bilateral stenting with radioactive seed placement provide equivalent safety and efficacy in the management of obstructive HC.

## INTRODUCTION

Hilar cholangiocarcinoma (HC) is a primary malignancy of the bile duct, frequently presenting with obstructive jaundice.^1^;^2^;^3^ Although curative options, such as resection or liver transplantation, are available,^4^ fewer than one-third of patients are candidates for surgery at diagnosis.^5^ Consequently, biliary stenting remains the first-line palliative strategy, as it provides rapid relief of obstruction and jaundice.^6^;^7^;^8^ However, long-term efficacy is often hindered by stent restenosis, with reported rates ranging from 28.6% to 39.4%.^9^ Tumor ingrowth is the predominant cause, highlighting the importance of integrating local tumor control with stenting to sustain long-term patency.

Placement of radioactive iodine ^125^I seed strands has emerged as an effective local therapy in several malignancies.^10^;^11^;^12^ Conventional computed tomography (CT)-guided direct implantation is well suited for solid, mass-like tumors, but less applicable to the intraluminal growth pattern of cholangiocarcinoma. To overcome this limitation, combined approaches thar involve embedding radioactive seeds alongside biliary stents have been developed.^7^;^8^

Another ongoing clinical debate concerns which type of stenting—unilateral or bilateral— provides better outcomes in HC. While numerous studies have examined the safety and efficacy of unilateral vs bilateral drainage using fluoroscopic or endoscopic guidance,^6^;^13^ most did not incorporate radioactive seed therapy. Thus, evidence directly comparing unilateral and bilateral stenting with radioactive seed placement for obstructive HC remains lacking.

## AIM

This study was aimed to compare the safety and therapeutic outcomes of unilateral and bilateral stenting with radioactive seed placement in patients with obstructive HC.

## MATERIALS AND METHODS

### Ethics

This retrospective analysis was performed under approvals from the Ethics Committees of the Xuzhou Mine Hospital (XZSKSYY2025-01) and the Xuzhou Central Hospital (XZXY-LK-20240111–018). Due to its retrospective design, the requirement for written informed consent was waived.

### Patient selection

From January 2022 to December 2024, consecutive patients diagnosed with obstructive HC who underwent stent implantation combined with radioactive seed strand placement at either the Xuzhou Central Hospital or the Xuzhou Mine Hospital were enrolled. The patients were assigned to either the unilateral or bilateral stent-with-seed groups, according to the procedure performed. Before treatment, we explained the details of both procedures to each patient. The patients chose the treatment option according to their economic condition. Eligibility criteria included: 1) confirmed diagnosis of HC, 2) presence of obstructive jaundice, and 3) eligibility for surgery. Exclusion criteria comprised: 1) Eastern Cooperative Oncology Group (ECOG) performance status above 2, 2) Bismuth type I HC, 3) concurrent malignancy, 4) prior hepatectomy, and 5) life expectancy under 3 months.

Diagnosis of obstructive HC was established using abdominal CT and / or magnetic resonance imaging in combination with intraductal biopsy. The patients were classified as inoperable if any of the following applied: 1) poor surgical tolerance, 2) presence of distant metastasis, and 3) extensive local invasion involving adjacent tissues or organs.

### Stent and radioactive seeds

Self-expanded stents (Micro-Tech, Nanjing, China) with a diameter of 8 mm and a length of 50–70 mm were employed for all procedures. Radioactive seed strands were manually prepared by encapsulating multiple [^125^I] seeds (0.8 mm × 4.5 mm) within a 4-F catheter, aligned in a straight configuration. The strand length was matched to that of the stent.

### Interventional approach

For unilateral stenting with seed placement, the right intrahepatic bile duct was punctured under local anesthesia using a 21-G Chiba needle (Cook, Bloomington, Indiana, United States) and fluoroscopic guidance. Contrast injection delineated the obstruction. A 6-F sheath (Cook) was then introduced, and a guidewire (Terumo, Tokyo, Japan) was advanced with the aid of a catheter (Cordis, Hialeah, Florida, United States) to traverse the obstruction. After introducing the catheter into the duodenum, it was exchanged for a stiff guidewire. The stent delivery system was advanced over this wire, and the radioactive seed strand was inserted through the 6-F sheath in parallel with the stent. The stent was deployed first, followed by careful sheath withdrawal, leaving the seed strand positioned between the stent and the bile duct wall.

For bilateral placement, both the right and left intrahepatic biliary ducts were accessed, and stents along with radioactive seed strands were deployed in parallel with one another.

### Follow-up

Postprocedural follow-up was performed at 2 weeks, 1 month, 3 months, 6 months, and every 6 months thereafter. Each evaluation included a physical examination, laboratory blood testing, and abdominal CT imaging.

### Assessments

Technical success was defined as the successful relief of biliary obstruction following radioactive seed stent placement without displacement of the seed strand.^5^ Clinical success was defined as a reduction in total bilirubin (TBIL) to below 70% of the preprocedural value within 2 weeks of stent implantation.^5^ Stent patency was measured from the time of stent insertion to either the recurrence of obstructive jaundice, patient death, or the last follow-up. Overall survival (OS) rate was calculated from the date of stent placement until death or the most recent follow-up.

The primary end point of this study was stent patency. Secondary outcomes included the OS rate, clinical success rate, technical success rate, and complication incidence.

### Statistical analysis

Continuous variables were reported as mean (SD) and compared via the *t *test if normally distributed, or reported as median (interquartile range [IQR]) and analyzed using the Mann–Whitney test when skewed. Changes in TBIL, alanine aminotransferase (ALT), and aspartate aminotransferase (AST) levels before and after intervention were examined with paired *t* tests or the Wilcoxon signed-rank tests. Categorical data were assessed using the χ² test. Stent patency and OS were analyzed using the Kaplan–Meier method and compared via the log-rank test. A 2-tailed *P *value below 0.05 was deemed significant. SPSS Statistics software, version 16.0 (IBM, Armonk, New York, United States) was used for all statistical testing.

## RESULTS

### Patients

**Figure 1:**
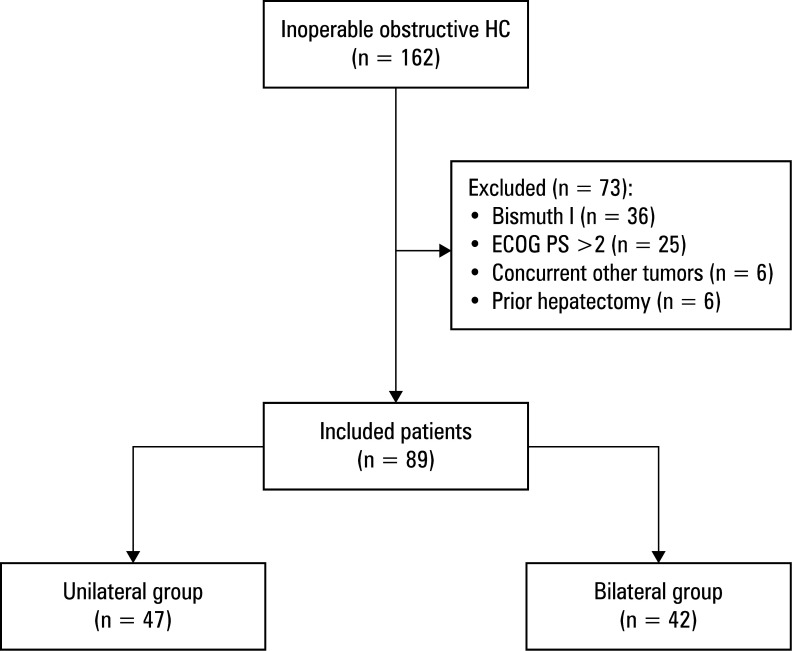
Flowchart of the study

Abbreviations: ECOG PS, Eastern Cooperative Oncology Group performance status; HC, hilar cholangiocarcinoma

**Figure 2:**
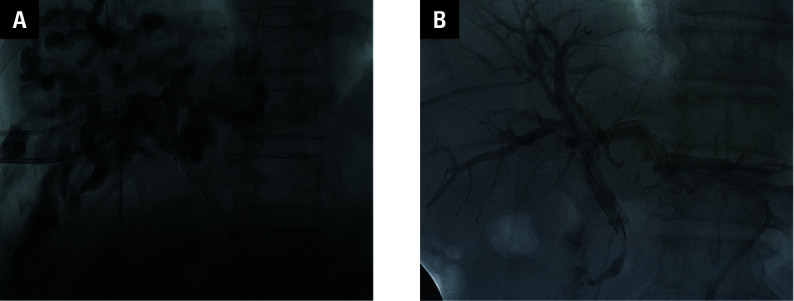
Fluoroscopy images of a unilateral (**A**) and bilateral (**B**) stent with radioactive seed placement

A total of 89 patients with obstructive HC were included in this study Figure 1: 47 underwent unilateral stent with radioactive seed placement (Figure 2 A) and 42 underwent bilateral placement (Figure 2 B). Baseline demographic and clinical parameters were well balanced between the 2 groups Table 1. All patients received postoperative chemotherapy.

**Table 1:** Baseline data of the patients

Parameter		Unilateral group (n = 47)	Bilateral group (n = 42)	*P *value
Age, y, mean (SD)/coefficient of dispersion		63.4 (8.9)/0.14	63.7 (8.9)/0.14	0.85
Sex, n (%)	Men	27 (57.4)	24 (57.1)	0.98
	Women	20 (42.6)	18 (42.9)	
Bismuth type, n (%)	II	19 (40.4)	19 (45.2)	0.89
	III	20 (42.6)	16 (38.1)	
	IV	8 (17)	7 (16.7)	
ECOG PS, mean (SD)/coefficient of dispersion		1.3 (0.6)/0.46	1.3 (0.6)/0.46	0.93
TBIL, μmol/l, median (IQR)/coefficient of dispersion	Before	189 (123–304)/0.55	173 (124–291)/0.5	0.68
	After	100 (36–117)/0.59	99 (31–116)/0.63	0.95
	*P* value^a^	<⁠0.001	<⁠0.001	–
AST, U/l, median (IQR)/coefficient of dispersion	Before	108 (86–199)/0.72	120 (86–208)/0.65	0.94
	After	50 (40–85)/0.6	50 (40–79)/0.69	0.94
	*P* value^a^	<⁠0.001	<⁠0.001	–
ALT, U/l, median (IQR)/coefficient of dispersion	Before	124 (76–257)/0.69	126 (65–250)/0.69	0.68
	After	54 (36–90)/0.62	58 (33–80)/0.69	0.95

**a ***P *value before and after treatment

Abbreviations: ALT, alanine aminotransferase; AST, aspartate aminotransferase; IQR, interquartile range; TBIL, total bilirubin; others, see Figure 1

### Technical success

The primary technical success rate was 91.5% in the unilateral group and 95.2% in the bilateral group (*P *= 0.68). Technical failure occurred in 6 patients (4 in the unilateral group and 2 in the bilateral cohort) due to inability to advance the guidewire across the stricture. These patients initially received temporary biliary drainage catheters, and definitive stent placement with radioactive seeds was successfully achieved 5 days later. Thus, the secondary technical success rate reached 100% in both groups Table 2.

**Table 2:** Treatment efficacy

Parameter	Unilateral group (n = 47)	Bilateral group (n = 42)	*P* value
Primary technical success	43 (91.5)	40 (95.2)	0.68
Secondary technical success	100	100	NA
Clinical success	42 (89.4)	38 (90.5)	>0.99
Stent restenosis	12 (25.5)	9 (21.4)	0.65
Stent patency, d, median (IQR)/coefficient of dispersion	220 (160–256)/0.43	210 (166–216)/0.37	0.79
Overall survival, d, median (IQR)/coefficient of dispersion	255 (178–369)/0.43	242 (175–362)/0.44	0.81

Data are presented as numbers (percentages) unless indicated otherwise.

Abbreviations: NA, not applicable; see Table 1

### Clinical success

Clinical success was achieved in 89.4% of the unilateral and 90.5% of the bilateral cases (*P* >0.99; Table 2). Pre- and postprocedural values of TBIL, ALT, and AST are summarized in Table 1. Among the 11 patients who did not meet the clinical success threshold (5 in the unilateral group and 6 in the bilateral cohort), TBIL levels decreased following the intervention but did not reach the predefined 70% reduction criterion.

### Stent patency

**Figure 3:**
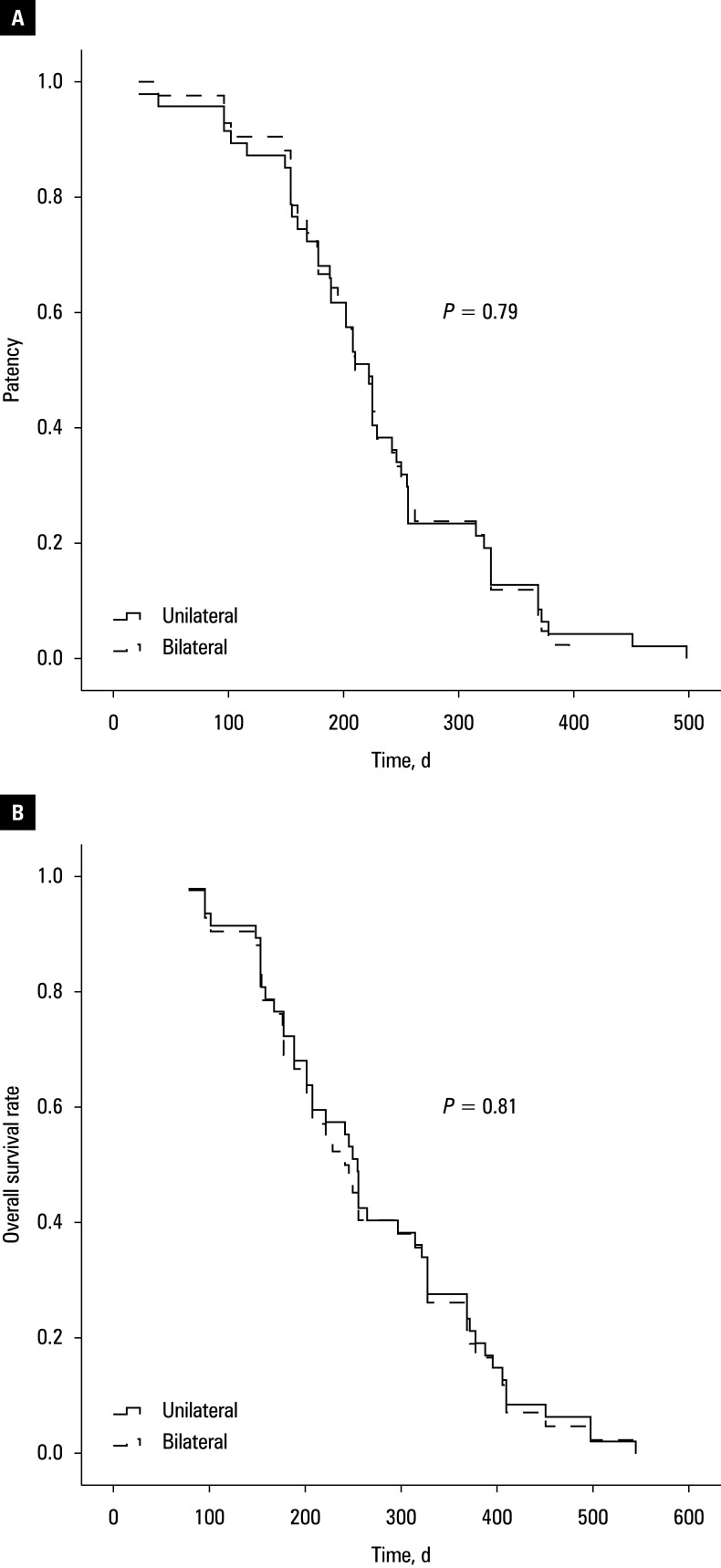
Stent patency (**A**) and overall survival rate (**B**) in the unilateral and bilateral groups

The rates of stent restenosis were 25.5% in the unilateral and 21.4% in the bilateral groups (*P* = 0.65; Table 2), with all cases attributed to tumor progression. In the unilateral group, restenosis was managed by contralateral stent insertion without radioactive seed placement in 8 patients (66.7%), and biliary drainage catheter placement in 4 patients (33.3%). In the bilateral group, all restenosis cases were managed with biliary drainage catheters. The stent reintervention rate was higher in the unilateral than the bilateral group (66.7% vs 0%; *P* = 0.005). Median (IQR) stent patency did not differ between the groups (220 [160–256] vs 210 [166–216] d; *P* = 0.79; Figure 3 A).

### Overall survival

**Figure 4:**
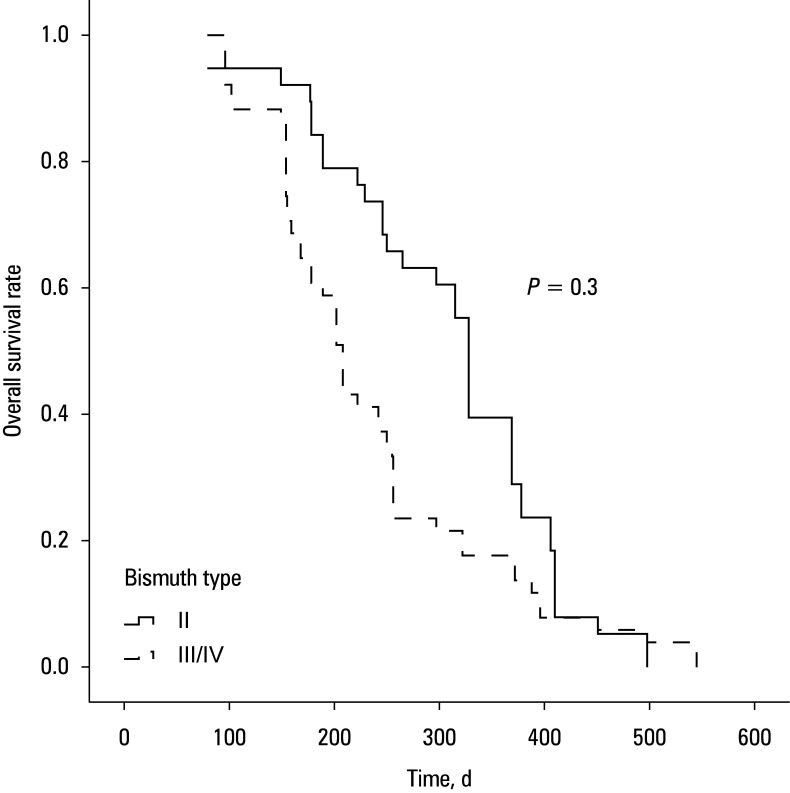
Overall survival rate in Bismuth II and III/IV patients

All patients died during follow-up, primarily due to tumor progression. Median (IQR) OS was 255 (178–369) days in the unilateral group and 242 (175–362) days in the bilateral group (*P* = 0.79; Figure 3 B). For all of the 89 patients, those with Bismuth type II disease had longer median OS, as compared with the individuals with type III/IV disease (328 vs 208 d; *P* = 0.03; Figure 4).

Figure description...

### Postoperative complications

The incidence of cholangitis was 10.6% in the unilateral group and 9.5% in the bilateral group (*P* >0.99), while bleeding occurred in 4.3% and 4.8% of the patients, respectively (*P* >0.99; Table 3). Thirty-day complication rates were also similar between the groups (8.5% vs 9.5%; *P* >0.99). Cholangitis was managed with intravenous antibiotics in 3 cases (2 in the unilateral cohort and 1 in the bilateral group) and percutaneous biliary drainage was performed in 6 cases (3 in each group). All bleeding events were successfully treated with hemostatic interventions.

**Table 3:** Postoperative complications

Complication	Unilateral group (n = 47)	Bilateral group (n = 42)	*P *value
Cholangitis	5 (10.6)	4 (9.5)	>0.99
Bleeding	2 (4.3)	2 (4.8)	>0.99
30-day complications	4 (8.5)	3 (9.5)	>0.99

Data are presented as numbers (percentages).

## DISCUSSION

Management of obstructive HC remains a considerable clinical challenge. Although biliary stenting is widely used to palliate jaundice and improve quality of life, several key issues continue to be debated. Conventional self-expanding metal stents provide mechanical relief of obstruction but have no intrinsic antitumor effect. To address this limitation, radioactive stents or stents combined with radioactive seed strands have been introduced, offering a dual benefit of luminal drainage and localized brachytherapy.^14^ Another unresolved issue concerns the optimal access route. Both percutaneous and endoscopic techniques are used in clinical practice, yet evidence from a meta-analysis suggests that percutaneous stent placement provides higher technical success rates and improved safety in patients with obstructive HC.^15^ Finally, the choice between unilateral and bilateral drainage has long been a subject of debate. Multiple meta-analyses have examined this question, but very few have focused specifically on stents incorporating radioactive seeds.

We explored the relative efficacy and safety of unilateral vs bilateral radioactive seed stent placement. Although ^5^ also conducted a similar study, the selection bias was significantly higher in their work because the patients in unilateral and bilateral groups were recruited in different time periods. In contrast, all patients in our study were from the same time period, which considerably diminished selection bias. Furthermore, our study was performed in 2 centers, whereas the study by ^5^ was a single-center anaysis.

Our technical success rates were high in both unilateral and bilateral groups (91.5% and 95.2%, respectively; *P* = 0.68), consistent with previous meta-analyses reporting success rates above 95% for conventional unilateral and bilateral biliary stenting.^16^ Several factors may account for these favorable results, such as performing all procedures under fluoroscopic guidance, ensuring accurate stent positioning, or deploying all stents percutaneously. This percutaneous biliary stenting approach has been successfully used as an alternative method in patients who did not undergo endoscopic biliary stent placement.^16^

Clinical success was also comparable between the 2 strategies, with both groups achieving effective resolution of jaundice and improvement in liver function. ^17^ suggested that drainage of at least 40% of functional liver volume was necessary to achieve clinical benefit. In our unilateral cohort, the stents were placed through the right intrahepatic approach, thereby preserving adequate functional liver remnant, which explains the satisfactory outcomes observed.

With respect to long-term outcomes, stent restenosis occurred in 25.5% of the unilateral and 21.4% of the bilateral cases (*P *= 0.65), with median stent patency of 220 and 210 days, respectively (*P* = 0.79). Although bilateral drainage theoretically offers greater durability by providing 2 drainage pathways, tumor progression remains the predominant cause of stent failure. Thus, tumor control, rather than the extent of biliary drainage, is the main determinant of stent longevity. The use of radioactive seed strands provides localized brachytherapy, which likely contributed to the relatively low and comparable restenosis rates in both groups.

An additional practical advantage of unilateral drainage is the feasibility of reintervention. In cases of restenosis, a contralateral intrahepatic approach can be used to place an additional stent. By contrast, revision following bilateral stenting is technically more challenging, limiting retreatment options when obstruction recurs.

OS was similar in both groups (255 vs 242 d; *P* = 0.79), which is consistent with the similarity in stent patency. These survival times are also in line with those reported in prior studies evaluating radioactive stent placement for HC, where median OS ranged from 220 to 256 days.^8^;^18^ Our analyses identified advanced Bismuth classification (type III/IV) and elevated postoperative AST levels as independent predictors of shorter survival. In line with these findings, the patients with Bismuth type II disease had considerably better survival rates than those with more advanced disease (328 vs 208 d; *P* = 0.03). The worse outcomes in Bismuth III/IV disease likely reflects more extensive tumor invasion, whereas a higher AST level possibly indicates impaired postoperative hepatic reserve that may be related to poorer survival.

Safety outcomes were favorable, with similar postoperative complication rates in both groups. Cholangitis and bleeding were infrequent, and overall 30-day complication rates (8.5% vs 9.5%) were consistent with previously reported ranges of 7.6%–12.6% for biliary stenting in HC.^19^;^20^ These results indicate that the choice between unilateral and bilateral placement does not substantially influence procedural safety.

Several limitations to this study should be noted. First, this was a retrospective study, and therefore subject to inherent selection bias. Nonetheless, baseline characteristics were comparable between the groups, which may have mitigated this concern. Second, the number of patients with Bismuth type IV disease was relatively small, limiting the statistical power for subgroup analysis. Third, due to the fact that the patients were recruited from 2 centers, procedural variations in operator experience and technical expertise could have introduced additional bias.

## CONCLUSIONS

In summary, this study demonstrates that unilateral and bilateral stenting with radioactive seed placement provide equivalent clinical efficacy, stent patency, OS, and safety in patients with obstructive HC. Given its procedural simplicity, feasibility of reintervention, and comparable outcomes, unilateral stenting may be the better approach.
